# Factors associated with geographic variation in cost per episode of care for three medical conditions

**DOI:** 10.1186/s13561-014-0008-4

**Published:** 2014-05-09

**Authors:** Jack Hadley, James D Reschovsky, James A O’Malley, Bruce E Landon

**Affiliations:** 1 Department of Health Administration and Policy, George Mason University, 4400 University Drive, MS 2G7 Fairfax, VA 22030, USA; 2 Mathematica Policy Research, 1100 1st Street, NE, 12th Floor, Washington, DC 20002-4221, USA; 3 The Dartmouth Institute and Geisel Medical School at Dartmouth, Dartmouth University, Lebanon, NH 03766, USA; 4 Department of Health Care Policy, Harvard University School of Medicine, 180 Longwood Avenue, Boston, Massachusetts 02115, USA

**Keywords:** Medicare reimbursement, Geographic variation in Medicare cost per episode, Physician supply

## Abstract

**Objective:**

To identify associations between market factors, especially relative reimbursement rates, and the probability of surgery and cost per episode for three medical conditions (cataract, benign prostatic neoplasm, and knee degeneration) with multiple treatment options.

**Methods:**

We use 2004*–*2006 Medicare claims data for elderly beneficiaries from sixty nationally representative communities to estimate multivariate models for the probability of surgery and cost per episode of care as a function local market factors, including Medicare physician reimbursement for surgical versus non-surgical treatment and the availability of primary care and specialty physicians. We used Symmetry’s Episode Treatment Groups (ETG) software to group claims into episodes for the three conditions (n = 540,874 episodes).

**Results:**

Higher Medicare reimbursement for surgical episodes and greater availability of the relevant specialists are significantly associated with more surgery and higher cost per episode for all three conditions, while greater availability of primary care physicians is significantly associated with less frequent surgery and lower cost per episode.

**Conclusion:**

Relative Medicare reimbursement rates for surgical vs. non-surgical treatments and the availability of both primary care physicians and relevant specialists are associated with the likelihood of surgery and cost per episode.

## Background

A recent study found little correlation across geographic areas in the cost per episode of treating ten different high prevalence and high cost conditions in the Medicare population [1]. Moreover, there was little consistency in the relationship between spending per episode for the different conditions and total Medicare spending per beneficiary in the area. Similarly, the Institute of Medicine concluded that geographic variations in cost per Medicare beneficiary do not reflect systematic variations across areas in how individual physicians treat particular conditions [2]. These reports suggest that general market characteristics, such as the per capita numbers of specialists, primary care physicians, and hospital beds do not have uniform effects on the cost of treating different conditions.

There are several potential reasons why cost per episode for particular conditions might vary across regions. First, physicians in some markets might have a lower threshold for diagnosing a particular condition. For instance, in the case of cataracts, late diagnosis could raise the average cost per episode because a higher proportion of cases might require surgery within a given year. Second, physicians in some markets might treat particular conditions more aggressively. For instance, for osteoarthritis of the knee, the threshold to offer knee replacement might vary across geographic areas. The reasons for these differences are not well understood, but may be influenced by a combination of local market conditions such as variations in specific reimbursement rates, the relative supplies of generalist and specialty physicians who can treat a particular condition, and differences in physicians’ approach to care.

Past research has documented regional variations in Medicare spending and the use of specific services per beneficiary [3-5], or costs and service use for people with specific illnesses [6-10]. Although some of the condition-specific studies examined correlations with the supplies of physicians or hospital beds in the area, they typically employed univariate comparisons, omitting potentially confounding variables. None of these studies investigated the role of area-specific reimbursement differences as a possible source of geographic variations.

One previous study investigated geographic variations (across thirteen metropolitan areas) in cost per episode for seven specific conditions and also found considerable inconsistencies in the relative costliness of different conditions within an area [11]. For example, compared to national average costs per episode, Minneapolis was 24% less costly for pneumonia and 28% more costly for coronary artery disease (CAD), while Miami was 34% less costly for CAD and 28% more costly for type-2 diabetes.

A more detailed comparison of cost per episode of CAD in Miami and Minneapolis suggested that different coding practices within the broad CAD designation might explain some of the difference in cost per episode. Specifically, physicians in Miami coded for a more narrow definition of CAD along with separate episodes of related conditions, while physicians in Minneapolis coded CAD under a broader definition that included related conditions that were coded separately in Miami. Accounting for differences in the number of episodes eliminated almost all of the cost difference, which shrank from $1,909 to $200 per person with a CAD episode. This analysis highlights the importance of using precisely defined clinical conditions for studying geographic variations in cost per episode.

To investigate whether condition-specific health care market factors, especially relative reimbursement rates for surgical and non-surgical treatments, are associated with geographic variation in the cost of treating episodes of different conditions, we analyzed three common conditions (cataract, benign prostatic neoplasm, and knee degeneration) characterized by substantial treatment choice discretion. The empirical analysis was guided by a conceptual framework that emphasizes the availability of specialists most closely associated with treating each condition, the availability of competing primary care physicians, and relative Medicare reimbursements for surgical and non-surgical treatments as factors that might influence treatment patterns.

## Methods

### Choice of conditions

The three conditions selected for analysis are prevalent in the Medicare population, are costly in aggregate, are treated by a mix of primary care and specific specialty physicians, and vary considerably in potential treatment aggressiveness. Each of these conditions is considered chronic and progressive if untreated. The three conditions also span various treatment settings: cataract surgery is outpatient; knee replacement surgery is almost exclusively an inpatient procedure; and treatment for benign prostatic neoplasm spans a range of surgical and non-surgical options.

Currently, there are no alternative treatments to cataract surgery. Rather, discretion related to cataract surgery depends on the threshold for offering or seeking surgical correction. Generally, surgery is offered when the cataract degrades vision to an extent that is noticeable by the patient, but there is no clearly defined threshold at which surgery should or should not be offered. Benign prostatic neoplasm can be treated by a variety of surgical approaches, which we combined into a single “surgery” category for analysis. Alternatives to surgery include no treatment and medical treatment, typically with alpha blockers (e.g., Flomax) or 5-alpha reductase inhibitors (e.g., finasteride). Effective surgical treatment for knee degeneration is limited to knee replacement, although in some circumstances, patients will be offered arthroscopic procedures to “clean out” the knee. Common alternatives include medication as well as joint injections with either steroids or products such as synvisc, which may temporarily help with symptoms. As with cataract, there is no clearly defined threshold for deciding when knee replacement surgery should be performed.

### Sample

We used claims data from a sample of elderly non-institutionalized Medicare beneficiaries without end-stage renal disease who were continuously enrolled in Parts A and B of the traditional fee-for-service program between 2004 and 2006, and who resided in sixty nationally representative communities from the Community Tracking Study (CTS) [12]. We included all Medicare claims of beneficiaries who received any billed service from a respondent to the 2004–2005 CTS Physician Survey at any time during the three-year period 2004–2006^a^. After exclusions, the full sample provided 4,448,612 annual observations. Details of the general sample design are provided elsewhere [13].

We used the Symmetry Episode Treatment Groups (ETG) software (Version 6) to group claims into episodes of care^b^. The program creates episodes by grouping clinically related services delivered to a patient for a given condition over a defined period of time, demarcated by “clean periods” of no service use for acute conditions, or by calendar years for chronic conditions. Although not developed specifically for Medicare, the limitations of using the Symmetry ETG grouper for a Medicare population are not particularly relevant to the three conditions we investigated and should be consistent across geographic areas [14].

We limited the sample to episodes that occurred in 2006 in order to use 2004–2005 claims to construct measures of episodes in prior years, coexisting medical conditions, and lagged information about local area diagnosis propensities and Medicare reimbursement rates. Episodes that started before January 1, 2006 or were still in progress by December 31, 2006 were eliminated from the analysis, regardless of their length. (Since episode start and end dates should be randomly distributed throughout the calendar year, this should not create any bias in the set of episodes analyzed.) We then limited the sample to episodes treated either by a relevant specialist or by a primary care physician (86.7% of cataract episodes, 91.3% of knee degeneration episodes, and 98.2% of benign prostatic neoplasm episodes). Episodes were attributed to the physician of either specialty who provided the most services for that particular episode.

### Dependent Variables

We constructed two dependent variables for each condition: whether surgery occurred during the episode and the total cost of the episode. Surgery was defined based on the presence of relevant surgical codes in the claims data. Total cost was calculated from the specific services assigned to the episode by the grouper after eliminating various adjustments the Medicare program makes to determine the amount it pays for each service. We constructed this measure of the “standardized” cost by:

 incorporating the full reimbursement from all payers (Medicare, patient cost sharing, and other insurers);

 eliminating geographic payment differences that account for local input price variation;

 eliminating differential payments for identical services across classes of providers (e.g. cost-based reimbursement for critical access hospitals vs. DRG-based payment for most hospitals); and

 distributing provider-specific special payments (e.g., disproportionate share and graduate medical education payments) across all hospitals.

(The Appendix describes details of the construction of the standardized cost variable).

### Independent variables

Independent variables measure personal and market characteristics that represent both the demand for care for each of the conditions as well as the supplies of relevant specialist and generalist physicians. The demand for care depends on the person’s health characteristics, income, and the ease/convenience (time price) of obtaining care. The supply of care to Medicare beneficiaries depends on the availability of both substitute and complementary resources, and the level of demand from people with other types of insurance coverage. Supply also depends on Medicare payment rates and on input prices, which are proxied by geographic fixed effects represented by dummy variables for each of the sixty CTS communities^c^.

#### Personal characteristics

To control for patients’ heterogeneous health states (casemix) at the time of treatment, all models include an extensive set of variables measuring individual patients’ co-existing health conditions drawn from the Hierarchical Condition Category (HCC) model [15]. Because we used standardized cost, not Medicare payment in our analysis, use of a single HCC score based on CMS weights (which change annually) would not be appropriate. Moreover, we chose to use concurrent casemix adjustment instead of prospective risk adjustment because the goal of this analysis is to investigate variations in current costs, not to predict future costs. Therefore, we included all of the variables used by the HCC model as regressors. (See Appendix). The HCC variables are those used by CMS at the time of our data—in 2006—and do not regularly change. We did not differentiate between community-based and institutionalized beneficiaries, as CMS risk adjustment does, because identification of institutionalized persons is not readily available in claims.

We also constructed dichotomous variables that measure whether the beneficiary had been treated for the particular study condition in prior years: no prior episodes in 2004 and 2005, episodes in both prior years, or in one or the other prior year. Interpretation of these variables is ambiguous because they could represent either long-term monitoring of a condition diagnosed relatively early in its clinical progression, or post-treatment monitoring of cases that have already received aggressive treatment.

Family income was imputed from a regression model estimated with data for elderly Medicare beneficiaries who responded to the 2003 CTS Household Survey [16]. The regression model, which is reported in Appendix, estimated self-reported income as a function of beneficiary age, gender, race, and population characteristics of the beneficiary’s zip code. We also include an indicator of whether the person was covered by Medicaid. Beneficiaries covered by Medicaid generally are not liable for any Medicare cost sharing. However, Medicaid programs in some states do not pay the full cost-sharing amount allowed by Medicare, so the potential effect of Medicaid coverage is ambiguous.

#### Market characteristics

These variables were defined for the county of the patient’s attributed physician because the attributed physician’s treatment decisions are most likely influenced by conditions in his/her local market, rather than the beneficiary’s county of residence. Potential competition from other physicians is measured by the numbers of physicians per 1,000 people in the specialty most closely associated with each of the study conditions (urology, orthopedic surgery, ophthalmology) per 1,000 people and in primary care practice (PCP). We hypothesize that the greater the availability of specialists, the more likely a person is to be treated by a specialist, which could be associated with a higher probability of surgery and a higher total cost per episode. Conversely, greater availability of PCPs could be associated with less frequent surgery and lower costs.

Since surgical treatment is one of the dependent variables, we include measures of hospital bed capacity (in the prostate and knee models) and ambulatory surgery center capacity (in the cataract and prostate models) per 1,000 people. We expect these to have positive associations with the likelihood of surgery and with total cost.

Medicare reimbursement rates have been shown to influence the supply of services. Prior studies have focused on whether the supply curve for physicians’ services is upward sloping, as predicted by standard economic theory, or backward bending, as would be predicted if income effects dominated. Although results are not entirely consistent, studies that tend to follow the comprehensive theoretical model developed by McGuire and Pauly [16], which allows for both types of behavior, were more likely to find a positive relationship between Medicare fees and the quantity of services provided to Medicare beneficiaries, though not necessarily across all types of services, specialties, or local practice situations [17-22].

Several earlier studies incorporating elements of the McGuire-Pauly model focused on Medicare supply responses to reductions in Medicare payments for selected services in the late 1980s. Escarce estimated an upward sloping supply response for services provided by physicians in five affected specialties, but mixed results in another study of eleven common surgical procedures [23,24]. Mitchell and Cromwell also found that results varied across specific surgical procedures, while Yip found that physicians whose incomes were most affected by lower Medicare fees for CABG did in fact increase their procedure volume [25,26].

In contrast, studies not based on the McGuire-Pauly framework found that Medicare fee changes either had no impact on volume or had an inverse relationship, i.e., supply increased in response to fee reductions [27,28]. Similarly, three earlier studies looking at associations between a 1977 Medicare fee change in Colorado and physicians’ Medicare service volume also found inverse relationships [29-31].

Differences in Medicare fees for similar services may also affect Medicare payments because of upcoding, i.e., billing for a more intensive level of care than what was actually supplied [32,33]. However, this type of behavior should not influence whether surgery was done unless it encourages outright fraud. Therefore, the consistency of the associations between relative Medicare fees and both the likelihood of surgery and the cost per episode will help interpret whether differences in costs are due only to upcoding or potentially also reflect differences in how episodes are treated.

We constructed a variable measuring relative Medicare reimbursement for physicians’ services as the ratio of average physician reimbursements for all surgical episodes to average physician reimbursements for all non-surgical episodes in the county in the two prior years (2004 and 2005). We hypothesize that this variable is a proxy for the relative amount of reimbursement a treating physician would expect to receive in 2006 for treating an average surgical case relative to an average non-surgical case. We used the county as the unit of geography rather than the Medicare payment area in order to capture variations in expected payment associated with variations in local treatment patterns. In effect, we assume that the average mix of services used to treat an episode is predetermined and that the physician’s decision is influenced by the expected payment for the local mix of services.

We recognize that this variable may be endogenous because it combines exogenous Medicare fees for individual services with average local treatment patterns. For example, physicians who have a preference for surgical treatment may choose a more expensive surgical procedure or may provide additional ancillary services in order to increase Medicare reimbursement for a surgical episode. If physicians with similar preferences tend to cluster in particular geographic areas, then the ratio of payments for surgical to non-surgical episodes may be positively correlated with the likelihood of surgery because of physicians’ preferences and their ability to influence reimbursement by selecting the mix of services that make up an episode of care.

To investigate this possible source of endogeneity bias we grouped counties into quartiles based on the ratio of the average Medicare payment per episode for surgical and non-surgical episodes of care, and compared the mixes of specific services provided in the 1^st^ and 4^th^ quartiles of counties. As shown in Appendix for cataract episodes, the mix of services for surgical episodes were generally similar in the two sets of areas. In fact, episodes in the counties in the lowest quartile of the payment ratio variable had somewhat greater percentages of higher cost services. Moreover, Medicare payments for both surgical and non-surgical episodes were higher in the counties in the 1^st^ quartile of the payment ratio than in the 4^th^ quartile counties, and the ratio of fees for specific services was not uniform, ranging from 1.43 to 1.00 for the surgical episodes. (Similar comparisons for non-surgical episodes showed that relative fees range from 1.35 to 1.12). Although this comparison does not provide clear evidence of a more expensive mix of services in areas with a high ratio of Medicare payments for surgical episodes compared to non-surgical episodes, we nonetheless test for possible endogeneity bias in the empirical estimation.

Early diagnosis of a condition can affect treatment decisions and episode cost if it is associated with a period of waiting for a condition to progress to the point when surgical treatment is appropriate. To control for this effect, we constructed measures of the local “propensity” to diagnose each condition. We first estimated a regression model of the probability of having an episode of a study condition over the full sample of Medicare beneficiaries over all three years as a function of age, gender, and race. We then used this model to predict the percentage of people in each county who should have the condition if the national rate of diagnosis, based on age, sex, and race, prevailed. We defined diagnosis propensity as the difference between the predicted and actual percentages of beneficiaries with each condition in the county.

Two additional market variables control for possible “spillover” effects of local market structure. One is the percentage of the Medicare population enrolled in Medicare Advantage plans in 2005. This could have either a positive or a negative effect on surgery and cost per episode depending on whether healthier beneficiaries select into Medicare Advantage or if the presence of Medicare Advantage plans induces less resource-intensive care in the local market. The second spillover variable is the percentage of the county population without health insurance^d^. We hypothesize that a high percentage uninsured increases physicians’ incentives to provide more services to Medicare beneficiaries. Finally, to control for unobserved variations in local input prices and market structure, all models include dummy variables to control for the specific CTS site and the local county’s position along the urban–rural continuum based on population size and proximity to a metropolitan statistical area.

### Statistical estimation

We estimated logistic regressions for the likelihood of surgery and linear regressions for standardized cost per episode. We also conducted two sensitivity tests to assess the robustness of the estimated Medicare reimbursement effect and its correlation with other variables in the model. First, we estimated the models excluding the Medicare reimbursement variable to assess whether the coefficients of the other key variables were sensitive to its inclusion. Second, we estimated the models limiting the sample to beneficiaries in counties that had at least ten surgical episodes in the prior time period, because the value of the reimbursement variable might be unstable if based on only a few surgical episodes in the county^e^. These models did not show substantive changes in the values of the parameter estimates, so we present results for the full sample.

We indicate the magnitudes of the associations between the key policy variables and the dependent variables by calculating elasticities from the cost models and marginal probabilities from the logistic models. Marginal probabilities were calculated by using the logistic regression models to predict the probability of a surgical episode first at the actual values of all of the independent variables, and then after increasing the values of the key policy variables by 10% in separate simulations.

## Results

### Descriptive characteristics

The final sample included 368,473 cataract episodes, 84,299 episodes of benign prostatic neoplasm, and 88,102 episodes of knee degeneration (Table 1). The percentage of surgical episodes ranges from 7.0% for benign prostatic neoplasm to 17.9% for cataracts. Average cost per episode for cataracts ($581.71) and benign prostatic neoplasm ($559.14) are fairly similar, but is much higher for knee degeneration ($3,260.03), and cost per episode is much higher for surgical than for non-surgical episodes (e.g., $2,711 vs. $118 for cataracts). The ratio of surgical to non-surgical Medicare physician payments per episode ranges from 9.66 (knee degeneration) to 13.36 (benign prostatic neoplasm). Area characteristics are similar across the three conditions.

**Table 1:** Mean values of dependent and selected independent variables, by condition

**Variable**	**Cataract**	**Knee degeneration**	**Benign neoplasm of the prostate**
EPISODE CHARACTERISTICS			
Number of episodes	368,473	88,102	84,299
Pct. surgical episodes	17.90	14.03	6.99
	(14.5-22.9)	(9.2-19.7)	(4.0-10.2)
Cost per episode ($)			
All episodes	581.71	3,260.03	559.14
	(492–724)	(2,326-4,172)	(410–711)
Non-surgical episodes	117.53	554.34	239.40
	(107–128)	(452–666)	(195–294)
Surgical episodes	2,710.97	19,834.43	4,811.77
	(2,494-2,984)	(15,580-22,670)	(4,323-5,507)
MARKET CHARACTERISTICS			
Ratio of Medicare physician payment per episode for surgical and non-surgical episodes	10.81	9.66	13.36
	(9.6-12.0)	(8.6-10.6)	(10.2-17.1)
Primary care practitioners per 1,000 pop.	0.77	0.81	0.78
	(0.52-1.01)	(0.53-1.07)	(0.51-1.05)
Relevant specialists per 1,000 pop.	0.08	0.10	0.05
	(0.04-0.11)	(0.06-0.14)	(0.02-0.06)
Ambulatory surgery center capacity per 1,000 pop.	0.05	--	0.05
	(0.02-0.08)		(0.02-0.09)
Hospital beds per 1,000 pop.	--	4.66	4.75
		(2.9-6.5)	(2.8-7.3)
Pct. Medicare pop. enrolled in Medicare advantage plans	12.14	12.73	13.49
	(0.1-35.6)	(0.2-35.9)	(0.2-35.9)
Pct. of the nonelderly pop. uninsured	16.26	16.28	16.60
	(10.9-22.9)	(10.9-22.9)	(10.7-23.6)
Diagnosis propensity (difference between actual and predicted proportion with diagnosis)	0.09	0.02	0.05
	(0.01-0.13)	(0.001-0.03)	(−0.001-0.08)
BENEFICIARY CHARACTERISTICS			
Family income ($1000 s)	40.89	40.31	52.07
	(26.0-56.1)	(25.1-54.4)	(32.5-70.6)
Pct. with medicaid coverage	7.64	8.83	4.80
	(2.9-20.4)	(3.5-18.8)	(1.1-14.9)
Pct. with episodes in prior years			
2004 & 2005	30.37	17.60	35.00
	(23.5-35.0)	(13.3-21.9)	(22.8-44.0)
2005 only	22.19	22.93	21.33
	(20.4-23.7)	(19.7-26.0)	(18.2-23.7)
2004 only	13.92	10.59	9.79
	(12.3-16.2)	(8.2-12.6)	(6.5-12.5)

(Site-level range between 10^th^-90^th^ percentiles in parentheses).

**Figure 1:**
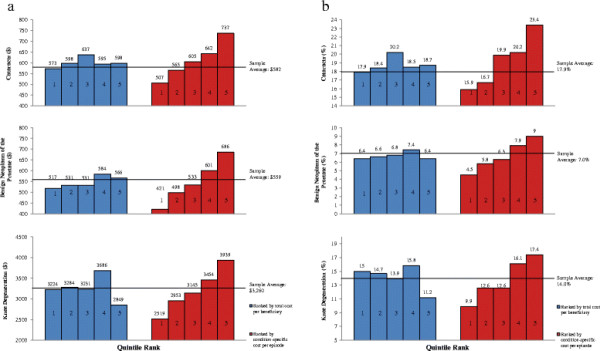
**Average cost per episode and percent surgical episodes by quintile site rankings for each condition.a.** Average cost per episode and **b.** Percent episodes treated by surgery.

Figure 1a and b compare the cost per episode and the percent of episodes treated surgically across areas (CTS sites) grouped into quintiles using two alternative ranking criteria: average total standardized Medicare cost per beneficiary for all care received (blue bars) and average standardized Medicare cost per episode for each of the three study conditions (red bars). When the sites are ranked by average total cost per beneficiary, there is little variation across quintiles in either cost per episode or the percentage of episodes treated surgically, and no discernible relationship between the average total cost per beneficiary and the cost per episode.

A very different picture emerges when sites are grouped into quintiles based on cost per episode for each condition. Cost per episode ranges from 45% to 63% higher in the fifth quintile compared to the first and the percentage of episodes treated surgically follows a very similar pattern, suggesting that the proportion treated surgically is a key driver of cost per episode.

### Regression results

To assess possible endogeneity bias of the lagged relative Medicare physician payment for surgical and non-surgical episodes, we conducted a Hausman-Wu test for each of the linear cost models [34]^f^. The exogenous identifying variable used to execute the test was the difference between the actual Medicare payment per relative value unit (RVU) for a typical service and a hypothetical payment per RVU that eliminates policy adjustments in the Medicare payment formula and uses more accurate local measures of input costs. This variable, constructed at the county level in another study [22], reflects the implicit profit per RVU due to deviations between Medicare’s actual payment rates and the estimated cost of a RVU.

The Hausman-Wu test rejected the presence of endogeneity bias in the models for benign prostatic neoplasm and knee degeneration, but failed to reject in the model for cataract episodes. We investigated instrumental variable estimation of the cataract model, but the resulting coefficient estimate had an extremely large standard error, perhaps reflecting the fact that the exogenous identifying variable is a generic measure of profit per RVU rather than specific to payments for cataract services. Thus, the OLS parameter estimate of the relative Medicare physician payment variable in the cataract episode model may be subject to endogeneity bias.

Table 2 reports the relative odds (probability of a surgical episode) and OLS coefficients (standardized cost per episode) from the regression models for selected variables. (Complete regression results for one of the conditions are reported in Appendix). Two sets of results are presented for each condition, one with the relative Medicare physician payment variable and one without.

**Table 2:** Regression results for probability of surgery and cost per episode

**Selected independent variables**	**Probability of surgery (Odds ratios)**		**Cost per episode (Regression coefficients)**	
	**Cataract**			
Medicare physician payment ratio	--	1.205	--	57.96
PCPs per 1000 pop.	0.730	0.665	−118.04	−147.99
Ophthalmologists per 1000 pop.	103.008	416.794	1927.73	2263.34
ASC capacity per 1000 pop.	18.115	12.998	731.66	559.47
Medicare HMO %	0.998	1.001 (.3)	−1.27(.02)	−0.87 (.10)
% Uninsured	1.030	1.036	9.77	11.74
Family income	0.998	0.998	−0.61	−0.59
County diagnosis propensity	0.574	0.343	−276.64	−521.37
Has Medicaid coverage	1.211	1.196	109.13	104.10
Episodes in 2005 & 2006	−0.262	−0.264	−151.81	−152.4
Episodes in 2004 & 2006	−0.375	−0.373	−215.27	−214.4
Episodes in 2004-2006	−0.461	−0.458	−237.47	−236.28
	**Knee degeneration**			
Medicare physician payment ratio	--	1.048	--	115.57
PCPs per 1000 pop.	0.814	0.821	−204.87 (.22)	−179.78 (.27)
Orthopedists per 1000 pop.	69.875	57.435	8608.65	8076.99
Hospital beds per 1000 pop.	0.981	0.979	−14.78 (.34)	−18.75 (.22)
Medicare HMO %	1.000 (.87)	1.001 (.31)	.67 (.92)	4.03 (.54)
% Uninsured	1.000 (.86)	1.003 (.29)	−14.50 (.29)	−8.32 (.54)
Family income	0.999	0.999	−2.67 (.02)	−2.636 (.02)
County diagnosis propensity	44.411	33.347	5783.25	5122.99
Has Medicaid coverage	0.554	0.553	−1050.76	−1058.45
Episodes in 2005 & 2006	0.608	0.608	1465.48	1465.09
Episodes in 2004 & 2006	0.282	0.282	492.64	492.75
Episodes in 2004-2006	0.315	0.315	681.79	681.77
	**Benign neoplasm of the prostate**			
Medicare physician payment ratio	--	1.022	--	8.57
PCPs per 1000 pop.	0.565	0.545	−174.1	−191.45
Urologists per 1000 pop.	372.571	595.574	2507.85	2720.77
Hospital beds per 1000 pop.	0.966	0.965	−15.59	−15.53
ASC capacity per 1000 pop.	6.910	8.129	573.2	647.11
Medicare HMO %	0.994	0.994	−1.48 (.25)	−1.25 (.33)
% Uninsured	1.035	1.034	14.04	14.25
Family income	0.998	0.998	−0.52	−0.52
County diagnosis propensity	0.080	0.059	−775.55	−920.16
Has medicaid coverage	1.432	1.436	231.25	232.14
Episodes in 2005 & 2006	−0.192	−0.193	−121.28	−121.53
Episodes in 2004 & 2006	−0.158	−0.158	−113.88	−113.52
Episodes in 2004-2006	−0.618	−0.617	−231	−230.46

Focusing first on the results for the probability of surgery, excluding relative Medicare payment from the models for knee degeneration and benign prostatic neoplasm has very little effect on the magnitudes of the other variables’ odds ratios, suggesting very little correlation with the other independent variables. However, the odds ratios in the cataract episode models show a greater sensitivity to excluding the Medicare payment variable.

The two measures of market demand characteristics, the percentage of the population without insurance and the percentage enrolled in a Medicare HMO, have no significant effect on the odds of a surgical episode for knee degeneration, but have generally significant but opposite associations with the other two conditions. In areas with large uninsured populations Medicare beneficiaries with cataract or a benign prostatic neoplasm are more likely to have a surgical episode. However, in areas with greater Medicare HMO enrollment, the odds of a surgical episode are lower. Family income, which is measured at the beneficiary level, has a consistently negative association with the odds of a surgical episode. This association could reflect either that higher income patients are diagnosed earlier in the course of their condition before surgical treatment is required, or that they had surgery more promptly (in an earlier year) than lower income people and were observed during a post-surgical episode. Having Medicaid coverage has a positive association with the probability of surgical treatment for cataracts and benign prostatic neoplasm, but a negative association with surgical treatment of knee degeneration.

The variable measuring county diagnosis propensity has similar associations with cataract and benign prostatic neoplasm, but an opposite association with knee degeneration episodes. For the latter, a high county-level diagnosis propensity is associated with a greater likelihood of a surgical episode. For the other two conditions, a high diagnosis propensity is associated with a lower likelihood of a surgical episode, suggesting that these conditions are diagnosed earlier in their course.

Turning to the models for cost per episode, the pattern of coefficient signs is very similar to those found in the logistic models. Given the strong association between cost per episode and the likelihood of surgery suggested by Figure 1a and b and the much higher cost of surgical episodes reported in Table 1, this result is not surprising. Levels of statistical significance tend to be lower, however, especially in the models for knee degeneration episodes. In particular, the number of PCPs per 1,000 population is not statistically significant, perhaps reflecting less substitutability between PCPs and specialists in the care of knee degeneration episodes.

### Magnitudes of associations

Table 3 summarizes the magnitudes of the associations (based on the regression results reported in Table 2) with the dependent variables for a 10% increase in the magnitudes of variables potentially amenable to policy intervention: primary care practitioners (PCPs) per 1,000 population, relevant specialists per 1,000 population, and the relative Medicare physician payment for surgical compared to non-surgical cases. (Although ambulatory surgery center and hospital bed capacity were also statistically significant, their marginal effects were very small and are not reported).

**Table 3:** Marginal probabilities and elasticities* of key policy variables: Percent change in dependent variable for a 10% **increase in the independent variable**

	**Cataract**	**Knee degeneration**	**Benign prostatic neoplasm**
**Percent surgical episodes (mean)**	17.9%	14.0%	6.99%
PCPs per 1000 pop.	−2.4% (−0.44%)	−1.2% (−0.18%)	−3.9% (−0.28%)
Relevant specialists per pop.	3.9 (0.7)	3.4 (0.48)	2.7 (0.18)
Medicare part B payment ratio	16.9 (3.04)	3.6 (0.51)	2.7 (0.19)
**Cost per episode (mean)**	$581.71	$3,260.03	$559.14
PCPs per 1000 pop.	−1.95%	−0.45%	−0.46%
Relevant specialists per 1000 pop.	3.00	2.54	3.90
Medicare part B payment ratio	10.77	3.42	2.05

*All calculated marginal probabilities and elasticities are based on coefficients that are statistically significant at p < .01. (See Table 2.)

(Absolute change in probability in parentheses).

For all three conditions, a 10% greater supply of primary care physicians is associated with a lower likelihood of surgery and lower cost per episode. For cataract episodes, for example, a 10% greater PCP supply is associated with 1.95% lower total cost per episode and 2.4% fewer surgical episodes. Conversely, a 10% greater supply of relevant specialists is associated with a higher total cost per episode and a greater likelihood of surgery, and this effect is generally larger than the association calculated for a change in PCP supply: estimated changes span a relatively narrow band of 2.54% to 3.9% across the three conditions.

Differences in Medicare physician reimbursement for a surgical episode relative to a non-surgical episode calculated at the county level are strongly associated with both the likelihood of surgery and standardized cost per episode. The physician payment ratio elasticity is largest for cataract episodes, and very similar in magnitude for knee degeneration and benign prostatic neoplasm episodes. A 10% higher level of physician reimbursement for cataract surgery relative to reimbursement for a non-surgical episode is associated with a higher surgical probability, by 16.9%, and a higher cost per episode, by 10.8%. For the other two conditions, the percentage increases associated with a 10% increase in physician reimbursement for surgical episodes range from 2.1% to 3.4%.

## Discussion

### Summary

Our study has several notable results. First, we find that average total spending per beneficiary for all services was not associated with cost per episode of the specific conditions we examined, and that the frequency of surgical versus non-surgical treatment in an area was strongly associated with the overall cost of treating that particular condition. Second, Medicare physician reimbursement for surgical relative to non-surgical episodes of care and the availability of specialists (ophthalmologists, urologists, and orthopedists) both have statistically significant and quantitatively meaningful positive associations with both the likelihood of surgical treatment and cost per episode. Third, the availability of PCPs has a significant negative association with both surgical treatment and cost, but the magnitude of the effect on cost is somewhat smaller than that for specialists. These results both refine and extend earlier findings of significant associations between the availabilities and supplies of relevant specialists and PCPs on the treatment and cost of particular conditions.

One potential explanation for some areas having a higher likelihood of surgical treatment and corresponding higher total costs per episode might relate to patterns of diagnosis in an area. For instance, in some areas, PCPs or other physicians might commonly diagnose and follow cataracts from their earliest stages, prior to the cataract compromising vision and requiring surgical treatment. In these areas, the prevalence of cataract episodes might be higher, but the average cost would be lower because surgery is required less frequently. In other parts of the country, this diagnosis might not be made or noted in claims until the condition has progressed and surgical options warrant consideration. The likelihood of surgery may be higher in these geographic areas because the cataract is diagnosed when more advanced. Thus, even though some markets might have higher rates of surgery and cost per episode, overall treatment costs for the condition might be lower because of the lower diagnostic propensity.

The statistical models included an area-level measure of diagnosis propensity to control for these effects. Notably, the relationships identified were highly statistically significant, but not consistent in direction across the three conditions: a high propensity to diagnose was negatively related to the probability of surgery and cost per episode for cataracts and benign prostatic neoplasm, but positively related to surgery and cost for knee degeneration. The reasons for these differences are not apparent, suggesting that analysis of variations in diagnosis propensity across areas and conditions should be a topic for future research.

Our analysis included variables measuring the availability of PCPs and specialists located the same county as the attributed physician, but excluded the actual specialty of the attributed physician. The decision to be treated by a specialist rather than a PCP very likely reflects differences in underlying severity of the condition that cannot be observed with claims data. Therefore, this analysis cannot reveal the mechanism through which greater PCP availability may affect the treatment of these episodes. One possibility is that greater availability of PCPs results in greater PCP involvement with these episodes before seeking a specialist’s opinion. This could influence whether and when surgical treatment is recommended. For example, PCPs’ involvement in episodes of knee degeneration and benign prostatic neoplasm may lead to more conservative treatment that emphasizes non-surgical interventions before concluding that surgery is needed.

We find that higher average Medicare physician payments for surgical episodes compared to non-surgical episodes are associated with a significantly greater likelihood of having a surgical episode and a higher average cost per episode. For knee degeneration and benign prostate episodes, this variable does not appear to be correlated with the supplies of relevant specialists or of PCPs. Thus, our findings are consistent with several earlier empirical studies that show that, at the margin, financial incentives to physicians lead to higher utilization of services. In this case, the higher relative reimbursement in these counties may lead to somewhat earlier and more frequent interventions for these conditions that could be treated either conservatively (without surgery) or more aggressively (with surgery).

The estimated effects of the relative payment variable are much larger for cataract episodes, and there is also stronger evidence of correlations with the supplies of physicians. Although these estimates may overstate the magnitude of the true relationship because of possible endogeneity bias, they are consistent with an earlier study of the effects of Medicare payments for cataract surgery, which found that a 10% increase in payment rates for cataract surgery increased the number of cataract surgeries performed by ophthalmologists by 11.5% [18].

### Limitations

This study has a number of important limitations. It is a cross-sectional analysis containing both supply and demand factors. Coefficients represent associations, not causal relationships between the dependent variables and relative Medicare reimbursements, specialty availability, and PCP availability. With respect to the relative Medicare reimbursement variable, the Hausman-Wu test suggests that it is likely endogenous in the case of cataracts, implying that relative Medicare reimbursement for surgery may be higher in areas where physicians perform more surgery because those physicians may be able to manipulate reimbursement rates to their advantage. Similarly, physicians with a preference for surgical intervention may choose to locate in areas where surgery is performed more frequently for some other reason we do not observe. Future analyses should attempt to treat both reimbursement rates and physician supply as potentially jointly determined with cost per episode and the probability of surgery in order to untangle the direction of causation.

Another limitation is that we investigate only three specific conditions. Therefore, our results do not generalize to all types of conditions and other specialties. Finally, while this analysis suggests how policy might influence the choice of treatment and episode cost, it does not address at all the potential benefits associated with earlier or more aggressive surgical treatment. Comparative effectiveness research is needed to address these questions.

## Conclusions

Although the cross-sectional structure of the analysis and the existence of potential endogeneity bias warrant caution in developing policy recommendations, we offer three tentative policy implications. First, efforts to alter the specialty mix of physicians by encouraging the expansion of primary care and discouraging entry into the specialties represented in this analysis could eventually affect the cost of treating these episodes by reducing the likelihood of surgical treatment. Moreover, policy should focus more precisely on the supplies of individual specialties thought to be associated with the over-provision of specific services in some areas, rather than on the supplies of broad categories of specialists, i.e., all surgical specialists or all medical specialists.

Second, modifying Medicare reimbursement rates for surgical procedures is another potential avenue for influencing the likelihood of surgical treatment and cost per episode. If it is believed that there is too much surgery, i.e., too many cases that do not benefit from surgery, then lowering Medicare payment rates for those procedures should reduce their volume and the average cost of treating episodes for these conditions.

Third, a policy focused on altering relative reimbursement rates for specific procedures has the advantage of rapid implementation and could affect all practitioners regardless of location. Even though changes in the supply of specialists and changes in Medicare relative payment rates have similar quantitative effects (Table 2), the cost and speed of implementing a change in reimbursement policy are much lower and faster than trying to use policy to change the relative supplies (and locations) of physicians in different specialties.

Although evidence is limited on how such reimbursement policies would affect treatment decisions, two studies of physicians’ responses to reductions in Medicare payment rates for treatment of prostate cancer patients found that physicians maintained treatment for clinically appropriate cases and reduced it for less appropriate cases [35,36]. Nonetheless, close and continuous monitoring of the effects on quality of changes in reimbursement rates is clearly important.

In conclusion, this analysis suggests that the choice of surgical treatment and cost per episode of care are associated with both market characteristics and relative reimbursement rates. However, the effects do not appear to be uniform across conditions or similar for all conditions across market areas. Therefore, policymakers would benefit from more clinically nuanced research studies that focus on specific conditions rather than on all medical care in general.

## Endnotes

^a^Center for Studying Health Systems Change. CTS Physician Surveys Details of the survey are available at http://www.hschange.org/index.cgi?data=04.

^b^Ingenix, Eden Prairie, MN.

^c^Although Medicare uses a fee schedule to pay providers uniform prices, imperfections in RVU assignments, geographic adjustments for local input prices, and other factors result in variations in effective fees levels across areas [22].

^d^U. S. Census Bureau, “2007 Health Insurance Coverage Status for Counties and States: Data Sets.” http://www.census.gov/did/www/sahie/data/20052007/index.html.

^e^In the full sample, cases in counties with fewer than 10 surgical episodes were assigned the CTS site-level value of this variable. The choice of 10 surgical episodes as the threshold for segmenting the sample is based on the observation that approximately half of the counties across the three conditions had fewer than 10 surgeries. However, these counties account for less than 5% of the total cases in the analysis.

^f^Endogeneity bias is less likely in the surgery models because the dependent variable is a dichotomous indicator that should be relatively insensitive to the mix of services provided to surgical and non-surgical episodes.

## Competing interests

The authors declare that they have no competing interests.

## Authors’ contribution

JH had primary responsibility for drafting the manuscript. All authors shared in the research design, the formulation of the statistical methods, and interpretation of the results. All authors read and approved the final manuscript.

## Appendix

The Appendix provides additional information about the construction of the dependent variable measuring the standardized cost per episode for each of the three conditions analyzed. Supplementary tables present the complete regression models with all independent variables for one of the conditions analyzed (knee degeneration), the regression model used to impute family income, and a comparison of the detailed service mix for cataract episodes in counties with high and low ratios of average Medicare payment for surgical and non-surgical cataract episodes of care.

## Construction of the “Standardized” cost per episode

This appendix describes the construction of the “standardized” cost measure used as the dependent variable in the analysis of cost per episode. The Medicare program bases its payments on a complex system of administered prices that in principle are designed to reflect the cost of local inputs, though in reality are modified to achieve other policy objectives. Our methods build upon and adapt procedures used by the Centers for Medicare and Medicaid Services in their development of resource use reports and MedPAC (2003). A key distinction between our measure of “standardized” cost and Medicare payments is that we measure total payments to providers for Medicare covered services rendered to Medicare beneficiaries, including not only payments from the Medicare trust funds, but also patient cost sharing and payments by other insurers. For instance, in the context of physician services, we base standardized costs on the total allowed charge for a given service, rather than just the Medicare payment.

## General adjustments

### Adjustments in payment based on the geographic location that service takes place

For nearly all types of services, Medicare adjusts payment levels to reflect local geographic variations in input prices such as labor, real estate costs, and other inputs to the production of medical services. In some cases, there are special rules that provide extra payment for rural providers and those who practice in designated provider shortage areas. Finally, for Part B services, some services are priced by Carriers. In constructing standardized prices we eliminate all of these geographic-based payment differences so that, for instance, a given service provided in New York City will receive the same standardized cost as one provided in rural Kansas, where wages and other input prices are generally less expensive.

### Adjustments in payments associated with different payment systems within a given class of providers

In some instances, Medicare payment policy identifies certain classes of providers for whom there are different payment systems than the norm. For example, while most short-term hospitals are paid prospectively on a DRG basis, rural Critical Access Hospitals (CAHs) are paid retrospectively on a cost basis. Moreover, Maryland hospitals are paid based on that state’s all-payer hospital rate setting system, rather than under regular DRG rules. Our standardized costs assigns a common cost to specific services regardless of whether or not the provider falls into a special class.

### Adjustments for provider specific differences in payment designed to achieve other social goals

In some cases, certain providers are eligible to receive add-ons to their Medicare payments by virtue of their case mix, function, or costs. Examples are the extra disproportionate share hospital (DSH) or graduate medical education (GME) payments that are paid to some hospitals. Under our procedures, for each specific hospital service, these extra payments are averaged out across all Medicare patients, regardless of which hospital they receive their care in.

## Adjustments for specific services

### Physician Services (except anesthesia)

For services with RVUs associated with them, the number of RVUs for each service (differentiating between provision in facility or nonfacility settings, as recorded in claims) was multiplied by the national conversion rate. Modifier codes that affect payment (but not those associated with HPSAs, etc.) and, where relevant, number of units, were incorporated into standardized costs. This procedure eliminates geographic adjustments. For carrier priced services that do not have RVU assignments, national means per HCPCS codes were assigned.

### Anesthesiology services

Standardized costs were based on national mean allowed charges by HCPCS code. This approach was used in large part because of complex rules regarding supervision of CRNAs by anesthesiologists, for which incomplete information was contained in claims files.

### Part B Drugs

Calculated as average national per unit payment made anytime in 2006 by HCPCS code multiplied by the number of units.

### Clinical Laboratory Services

Standardized costs were calculated as the National Limit Allowance (NLA) associated with each clinical lab HCPCS code. This standardized geographic variations across carriers. Nationally, nearly all clinical lab services are paid at NLA levels.

### Ambulance services

Assigned average allowed charge by ambulance HCPCS code, which adjusts for both payment differences across payment areas, rural add-on payments, and geographic differences in the average distances traveled.

### Community-based Ambulatory Surgical Centers

Based on HCPCS code and location of service, services were assigned the 2006 national APC conversion factor times the APC relative weight, with adjustments for modifiers.

### Hospital short-term acute inpatient services

Standardized costs were based on national average payment per DRG, with adjustments for transfers. No differentiation is made for CAHs or Maryland hospitals, whether the hospital received DSH or GME payments, or hospitals qualifying for bad debt adjustments.

### Long-term care hospitals

Standardized costs were based on the 2006 long-term care national base rate times the LTC-DRG relative weight.

### Inpatient rehabilitation facilities

The standardized cost was based on the mean national payment per CMG (case mix group).

### Skilled nursing facilities

We assigned the mean national per diem payment per RUG (Resource Use Group) score times the length of stay. Standardized costs eliminated the differential payment levels for urban and rural SNFs, as well as swing beds in CAHs.

### Home health agencies

We assigned 2006 national average cost per HHRG (home health resource group) for claims based on HHRGs. When the number of visits in the episode was less than five, standardized costs were based on the sum of nationally set (i.e. before geographic adjustments), per visit amounts associated with the type of visits listed in the claim, consistent with payment rules.

### Hospital outpatient services paid under the outpatient prospective payment system (OPPS)

Services paid under OPPS were assigned the relevant APC value (conversion value times the APC relative weight). Payment discounts for multiple procedures were made. No hold harmless payment adjustments were made for cancer, children’s or small rural hospitals and no special adjustments were made for CAHs, Indian Health Service facilities, or facilities in Maryland.

### Hospital outpatient services not covered by OPPS

Standardized costs were based on the mean national payment per HCPCS code, with adjustments made for number of units and modifiers where applicable. No differentiation is made between hospital based and freestanding facilities contained in the outpatient claims files for equivalent services.

### Durable Medical Equipment

Standardized costs were assigned as the average national payment by HCPCS code-modifier combination. Modifiers account for new vs. used equipment and whether the equipment was rented or purchased. Standardized costs account for the number of units, where relevant.

## Complete regression models for knee degeneration episodes

This section reports the complete regression models with all independent variables used to estimate the likelihood of a surgical episode (logistic regression) and the cost per episode (linear regression). Regression models for the other conditions are identical except for the variable measuring the supply of the relevant specialists, ophthalmologists for cataract episodes and urologists for benign neoplasm of the prostate. The variables that indicate the CTS site, individual’s demographic characteristics, and the presence of specific medical conditions are all dichotomous (Table 4).

**Table 4:** Complete regression models for knee degeneration episodes

	**Likelihood of a Surgical Episode**				**Cost per Episode**			
	**w/ Medicare**		**w/o Medicare**		**w/ Medicare**		**w/o Medicare**	
**Label**	**Odds Ratio**	**Pr>chi-sq**	**Odds Ratio**	**Pr>chi-sq**	**Param.**	**Pr > /t/**	**Param.**	**Pr > /t/**
Intercept		<.01		<.01	263.75	0.69	1481.86	0.01
Medicare relative payment	1.048	<.01			115.57	0.00		
Total hospital beds per 1,000 pop.	0.979	<.01	0.981	<.01	−18.75	0.22	−14.78	0.34
Medicare advantage %	1.001	0.31	1	0.88	4.03	0.54	0.67	0.92
Percent uninsured	1.003	0.29	1	0.85	−8.32	0.54	−14.50	0.29
Predicted family income, $1,000	0.999	<.01	0.999	<.01	−2.64	0.02	−2.67	0.02
PCPs per 1,000 pop	0.821	<.01	0.814	<.01	−179.78	0.27	−204.87	0.21
Orthopedic Surgeons per 1,000 pop	57.435	<.01	69.785	<.01	8076.99	<.01	8608.65	<.01
Knee diagnosis propensity	33.347	<.01	44.411	<.01	5122.99	0.00	5783.25	0.00
Knee in 2006 & 2005	1.837	<.01	1.837	<.01	1465.09	<.01	1465.48	<.01
Knee in 2006 & 2004	1.326	<.01	1.326	<.01	492.75	<.01	492.64	<.01
Knee in all 3 years	1.37	<.01	1.37	<.01	681.77	<.01	681.79	<.01
250,000 to 1 million pop.	1.113	<.01	1.101	<.01	248.86	0.16	218.53	0.22
Fewer than 250,000 pop.	1.092	0.01	1.018	0.60	−21.05	0.91	−178.17	0.33
Urban pop 20,000+, adjacent metro area	0.828	<.01	0.843	<.01	−515.46	0.01	−472.31	0.02
Urban pop 20,000+, not adjacent metro area	0.841	<.01	0.807	<.01	−804.99	0.00	−899.08	0.00
Completely rural or <20,000 urban pop, adjacent to a metro area	0.599	<.01	0.618	<.01	−1086.23	<.01	−1012.13	<.01
Completely rural or <20,000 urban pop, not adjacent to a metro area	0.835	<.01	0.841	<.01	−421.66	0.17	−390.97	0.20
Boston	1.999	<.01	1.977	<.01	459.11	0.30	424.87	0.33
Cleveland	1.862	<.01	1.844	<.01	−68.19	0.86	−92.57	0.81
Greenville	1.875	<.01	1.824	<.01	270.05	0.53	225.93	0.60
Indianapolis	2.289	<.01	2.331	<.01	481.85	0.28	521.84	0.24
Lansing	4.335	<.01	4.167	<.01	1743.84	0.00	1661.43	0.00
Little Rock	3.353	<.01	3.439	<.01	2073.97	<.01	2150.04	<.01
Newark	1.695	<.01	1.626	<.01	962.48	0.03	863.51	0.05
Orange Country	1.553	<.01	1.519	<.01	441.55	0.23	385.74	0.29
Phoenix	2.615	<.01	2.66	<.01	1245.15	0.00	1286.09	<.01
Seattle	4.234	<.01	4.1	<.01	1926.81	<.01	1843.13	<.01
Syracuse	1.866	<.01	1.816	<.01	196.01	0.67	137.73	0.77
Atlanta	1.926	<.01	1.841	<.01	439.34	0.29	331.66	0.43
Augusta	1.64	<.01	1.613	<.01	−105.73	0.82	−141.47	0.76
Baltimore	2.499	<.01	2.393	<.01	1444.44	0.00	1341.52	0.00
Bridgeport	1.783	<.01	1.74	<.01	492.16	0.27	440.17	0.33
Chicago	2.211	<.01	2.242	<.01	1086.10	0.01	1123.08	0.01
Columbus	1.524	<.01	1.55	<.01	−170.76	0.69	−127.22	0.77
Denver	2.251	<.01	2.323	<.01	782.53	0.03	860.28	0.02
Detroit	2.612	<.01	2.521	<.01	1042.65	0.02	965.72	0.03
Greensboro	2.366	<.01	2.34	<.01	737.38	0.06	713.82	0.06
Houston	2.297	<.01	2.387	<.01	769.28	0.05	871.75	0.02
Huntington	3.268	<.01	3.301	<.01	1215.06	0.01	1253.16	0.01
Killeen	1.061	0.59	1.218	0.07	−1071.01	0.02	−731.35	0.12
Knoxville	2.539	<.01	2.529	<.01	732.56	0.08	735.08	0.08
Las Vegas	2.538	<.01	2.644	<.01	1681.40	<.01	1782.93	<.01
Los Angeles	1.979	<.01	1.963	<.01	698.34	0.01	679.55	0.02
Middlesex	1.516	<.01	1.546	<.01	224.06	0.59	263.60	0.53
Milwaukee	2.711	<.01	2.671	<.01	928.57	0.04	895.44	0.05
Minneapolis	4.178	<.01	4.177	<.01	1422.31	0.00	1414.78	0.00
Modesto	3.488	<.01	3.802	<.01	1289.36	0.01	1502.17	0.00
Nassau	1.637	<.01	1.532	<.01	717.10	0.06	556.12	0.15
New York City	1.139	0.19	1.061	0.55	186.82	0.64	−13.89	0.97
Philadelphia	1.912	<.01	1.869	<.01	565.01	0.14	503.24	0.19
Pittsburgh	1.879	<.01	2.009	<.01	392.91	0.31	544.60	0.16
Portland	2.578	<.01	2.774	<.01	620.25	0.13	790.23	0.05
Riverside	2.819	<.01	2.929	<.01	1537.83	<.01	1634.94	<.01
Rochester	2.027	<.01	2.107	<.01	235.60	0.55	319.40	0.42
San Antonio	2.063	<.01	2.147	<.01	659.56	0.09	758.39	0.05
San Francisco	1.654	<.01	1.713	<.01	−346.54	0.39	−267.43	0.51
Santa Rosa	1.5	<.01	1.554	<.01	−245.71	0.55	−154.53	0.70
Shreveport	1.612	<.01	1.598	<.01	3.19	0.99	−8.20	0.99
St. Louis	3.115	<.01	3.05	<.01	1095.28	0.01	1045.82	0.01
Tampa	3.074	<.01	3.156	<.01	1547.16	<.01	1613.21	<.01
Tulsa	3.637	<.01	3.587	<.01	1648.34	0.00	1618.42	0.00
Washington DC	2.247	<.01	2.117	<.01	833.68	0.04	691.44	0.09
W Palm Beach	1.299	<.01	1.258	<.01	107.93	0.73	33.26	0.91
Worcester	1.426	<.01	1.441	<.01	−279.29	0.56	−259.90	0.59
Dothan	1.252	0.03	1.235	0.05	−0.29	1.00	−47.74	0.92
Terre Haute	1.936	<.01	1.948	<.01	689.23	0.17	635.20	0.20
Wilmington	2.695	<.01	2.631	<.01	1286.99	0.00	1238.71	0.00
W-Cen Alabama	5.728	<.01	5.735	<.01	1912.34	0.62	1917.45	0.62
Cen Arkansas	2.858	<.01	2.911	<.01	1460.14	0.00	1512.32	0.00
N Georgia	2.651	<.01	2.61	<.01	1258.66	0.00	1224.33	0.00
NE Illinois	4.713	<.01	4.993	<.01	2024.37	<.01	2185.34	<.01
NE Indiana	2.523	<.01	2.406	<.01	844.72	0.11	738.15	0.16
E Maine	4.272	<.01	4.149	<.01	2005.49	<.01	1935.89	<.01
E North Car	2.774	<.01	2.856	<.01	1440.15	0.00	1517.89	0.00
N Utah	3.588	<.01	3.746	<.01	2207.68	<.01	2297.29	<.01
NW Washington	5.126	<.01	4.948	<.01	2571.48	<.01	2483.29	<.01
Female, 70-74	0.869	<.01	0.868	<.01	−398.46	<.01	−398.37	<.01
Female, 75-79	0.77	<.01	0.77	<.01	−600.39	<.01	−600.47	<.01
Female, 80-84	0.604	<.01	0.604	<.01	−1092.32	<.01	−1094.17	<.01
Female, 85-89	0.301	<.01	0.3	<.01	−1923.30	<.01	−1927.73	<.01
Female, 90-94	0.081	<.01	0.081	<.01	−2879.62	<.01	−2878.14	<.01
Female, 95+	0.086	<.01	0.086	<.01	−2583.31	<.01	−2584.45	<.01
Male, 65-69	1.086	<.01	1.087	<.01	177.61	0.10	179.50	0.09
Male, 70-74	0.957	0.02	0.957	0.02	−242.75	0.02	−241.39	0.02
Male, 75-79	0.826	<.01	0.827	<.01	−446.79	<.01	−444.44	<.01
Male, 80-84	0.591	<.01	0.592	<.01	−1153.62	<.01	−1152.08	<.01
Male, 85-89	0.34	<.01	0.34	<.01	−2078.91	<.01	−2078.07	<.01
Male, 90-94	0.084	<.01	0.084	<.01	−2993.29	<.01	−2990.22	<.01
Male, 95+	0.011	<.01	0.011	<.01	−3829.36	<.01	−3822.73	<.01
Female covered by Medicaid	1.327	<.01	1.328	<.01	501.49	0.02	506.88	0.02
Covered by Medicaid	0.554	<.01	0.553	<.01	−1050.76	<.01	−1058.45	<.01
Originally disabled dummy variable	0.844	<.01	0.842	<.01	−215.49	0.03	−219.15	0.03
Diabetes and congestive heart failure	1.003	0.93	1.003	0.93	−38.62	0.81	−37.29	0.82
Diabetes and cardiovascular disease	1.075	0.12	1.075	0.12	105.11	0.66	106.14	0.66
Congestive heart failure and COPD	0.495	<.01	0.496	<.01	−1754.30	<.01	−1752.41	<.01
COPD, cardiovascular disease, and coronary artery disease	0.675	<.01	0.674	<.01	−964.91	0.04	−968.02	0.04
Arthritis and congestive heart failure	0.878	0.02	0.877	0.01	−461.98	0.09	−465.22	0.09
Arthritis, congestive heart failure, and diabetes	0.835	<.01	0.835	<.01	−597.90	0.03	−599.91	0.03
HIV/AIDS	0.847	0.58	0.848	0.58	−1015.23	0.47	−1022.80	0.46
Septicemia/Shock	0.637	<.01	0.635	<.01	−686.78	0.01	−690.87	0.01
Opportunistic Infections	0.724	0.00	0.716	<.01	−543.88	0.31	−559.52	0.29
Metastatic Cancer and Acute Leukemia	0.487	<.01	0.486	<.01	−1506.89	<.01	−1509.66	<.01
Lung, Upper Digestive Tract, and Other Severe Cancers	0.661	<.01	0.66	<.01	−857.56	0.00	−863.01	0.00
Lymphatic, Head and Neck, Brain, and Other Major Cancers	0.761	<.01	0.761	<.01	−568.29	0.00	−568.29	0.00
Breast, Prostate, Colorectal and Other Cancers and Tumors	0.931	<.01	0.93	<.01	−144.57	0.06	−145.97	0.06
Diabetes with Renal or Peripheral Circulatory Manifestation	0.965	0.23	0.964	0.22	188.89	0.23	183.82	0.24
Diabetes with Neurologic or Other Specified Manifestation	1.061	0.03	1.06	0.03	230.57	0.12	229.68	0.12
Diabetes with Acute Complications	1.236	0.03	1.227	0.03	585.17	0.25	567.65	0.26
Diabetes with Ophthalmologic or Unspecified Manifestation	0.731	<.01	0.729	<.01	−487.34	0.01	−493.09	0.01
Diabetes without Complication	1.049	<.01	1.049	<.01	125.82	0.06	126.91	0.05
Protein-Calorie Malnutrition	1.642	<.01	1.638	<.01	1346.17	<.01	1345.01	<.01
End-Stage Liver Disease	1.05	0.64	1.053	0.63	525.14	0.37	530.48	0.36
Cirrhosis of Liver	0.978	0.80	0.975	0.78	291.17	0.53	279.44	0.55
Chronic Hepatitis	1.011	0.90	1.015	0.87	−47.26	0.92	−42.97	0.93
Intestinal Obstruction/Perforation	0.905	<.01	0.905	<.01	−667.95	0.00	−667.78	0.00
Pancreatic Disease	0.657	<.01	0.657	<.01	−1061.98	<.01	−1065.62	<.01
Inflammatory Bowel Disease	1.054	0.22	1.053	0.23	−146.24	0.54	−147.79	0.54
Bone/Joint/Muscle Infections/Necrosis	2.468	<.01	2.466	<.01	3429.72	<.01	3427.43	<.01
Rheumatoid Arthritis and Inflammatory Connective Tissue Disease	0.917	<.01	0.917	<.01	−163.11	0.05	−163.96	0.05
Severe Hematological Disorders	1.057	0.21	1.059	0.19	82.79	0.72	85.35	0.71
Disorders of Immunity	1.079	0.10	1.079	0.10	212.16	0.38	216.60	0.37
Drug/Alcohol Psychosis	4.755	<.01	4.765	<.01	6377.99	<.01	6379.30	<.01
Drug/Alcohol Dependence	1.443	<.01	1.449	<.01	866.60	0.08	878.27	0.08
Schizophrenia	0.532	<.01	0.531	<.01	−672.90	0.23	−683.61	0.22
Major Depressive, Bipolar, and Paranoid Disorders	0.995	0.84	0.993	0.79	79.90	0.57	77.78	0.58
Quadriplegia, Other Extensive Paralysis	0.579	0.00	0.577	0.00	−1305.80	0.08	−1312.71	0.08
Paraplegia	1.295	0.14	1.292	0.15	4976.07	<.01	4972.00	<.01
Spinal Cord Disorders/Injuries	0.754	<.01	0.753	<.01	−665.65	0.02	−665.73	0.02
Muscular Dystrophy	0.455	0.19	0.452	0.19	−2069.78	0.38	−2089.42	0.37
Polyneuropathy	1.067	<.01	1.067	0.00	331.19	0.00	331.35	0.00
Multiple Sclerosis	0.632	<.01	0.629	0.00	−975.37	0.09	−988.91	0.08
Parkinsons and Huntingtons Diseases	0.852	<.01	0.853	0.00	258.96	0.25	265.40	0.24
Seizure Disorders and Convulsions	1.101	0.01	1.102	0.01	42.32	0.83	44.85	0.82
Coma, Brain Compression/Anoxic Damage	1.168	0.13	1.169	0.13	−319.24	0.55	−314.72	0.55
Respirator Dependence/Tracheostomy Status	0.433	<.01	0.442	0.00	−1904.37	0.04	−1888.25	0.04
Respiratory Arrest	1.291	0.13	1.289	0.14	982.22	0.26	976.57	0.27
Cardio-Respiratory Failure and Shock	2.12	<.01	2.136	<.01	2272.11	<.01	2288.18	<.01
Congestive Heart Failure	1.016	0.46	1.014	0.50	34.53	0.74	32.35	0.76
Acute Myocardial Infarction	0.698	<.01	0.696	<.01	−1073.10	<.01	−1079.00	<.01
Unstable Angina and Other Acute Ischemic Heart Disease	1.051	0.05	1.052	0.05	211.42	0.12	214.37	0.12
Angina Pectoris/Old Myocardial Infarction	1.478	<.01	1.476	<.01	866.16	<.01	864.08	<.01
Specified Heart Arrhythmias	1.078	<.01	1.079	<.01	180.90	0.01	183.43	0.01
Cerebral Hemorrhage	0.726	<.01	0.729	0.00	−322.67	0.42	−317.69	0.43
Ischemic or Unspecified Stroke	0.931	0.02	0.93	0.02	−171.89	0.26	−173.23	0.26
Hemiplegia/Hemiparesis	0.785	<.01	0.785	0.00	−397.62	0.21	−400.07	0.21
Cerebral Palsy and Other Paralytic Syndromes	1.543	<.01	1.537	0.00	1015.38	0.15	1009.78	0.16
Vascular Disease with Complications	1.152	<.01	1.152	<.01	456.53	0.00	456.91	0.00
Vascular Disease	1.307	<.01	1.307	<.01	741.59	<.01	741.82	<.01
Cystic Fibrosis	0.07	<.01	0.07	0.00	−3123.65	0.067	−3148.35	0.07
Chronic Obstructive Pulmonary Disease	1.498	<.01	1.495	<.01	972.38	<.01	968.82	<.01
Aspiration and Specified Bacterial Pneumonias	0.555	<.01	0.551	<.01	−1123.07	0.00	−1135.55	0.00
Pneumococcal Pneumonia, Emphysema, Lung Abscess	0.868	0.11	0.865	0.10	−370.29	0.42	−372.75	0.42
Proliferative Diabetic Retinopathy and Vitreous Hemorrhage	0.602	<.01	0.601	<.01	−1144.23	0.00	−1143.37	0.00
Dialysis Status	0.603	0.02	0.599	0.01	−1295.52	0.24	−1308.01	0.24
Renal Failure	1.263	<.01	1.263	<.01	548.15	<.01	549.32	<.01
Nephritis	1.531	<.01	1.53	<.01	1521.63	0.00	1519.22	0.00
Decubitus Ulcer of Skin	1.896	<.01	1.891	<.01	2250.55	<.01	2244.65	<.01
Chronic Ulcer of Skin, Except Decubitus	0.904	<.01	0.903	0.00	−53.72	0.71	−55.19	0.70
Extensive Third-Degree Burns	0.277	0.21	0.271	0.20	−1731.37	0.63	−1759.98	0.63
Severe Head Injury	0.581	0.31	0.58	0.31	−732.45	0.78	−720.63	0.78
Major Head Injury	0.796	<.01	0.798	0.00	−953.25	0.01	−952.89	0.01
Vertebral Fractures without Spinal Cord Injury	0.671	<.01	0.671	<.01	−746.57	<.01	−750.99	<.01
Hip Fracture/Dislocation	0.752	<.01	0.752	<.01	−622.68	0.00	−621.20	0.00
Traumatic Amputation	7.719	<.01	7.699	<.01	7341.31	<.01	7337.40	<.01
Amputation, Lower Limb/Amputation Complications	8.228	<.01	8.202	<.01	8871.35	<.01	8868.30	<.01
Major Complications of medical Care and Trauma	4.163	<.01	4.167	<.01	5813.92	<.01	5817.42	<.01
Major Organ Transplant Status	0.545	<.01	0.545	0.00	−1045.99	0.24	−1059.98	0.24
Artificial Openings for Feeding or Elimination	1.063	0.39	1.065	0.37	656.54	0.10	659.92	0.10

## Regression model used to impute family income

This model was estimated using data for 5,554 elderly respondents to the 2003 Community Tracking Study Household Survey. The dependent variable was the natural log of family income, which was converted to natural numbers using a smearing adjustment. The model also includes site dummies for the 60 CTS sites (coefficients not shown). Zip code area characteristics are from the 2000 Census. The parameters of this model, which has a R^2^=0.25, were applied to the similar characteristics of patients in each of the condition episodes to impute a value of family income (Table 5).

**Table 5:** Family Income Imputation Model

**Variable**	**Coefficient**	**P-Value**
Intercept	10.02	<.01
Age 70–74 (ref. group = age 65–69)	−0.14	<.01
Age 75-79	−0.21	<.01
Age 80-84	−0.40	<.01
Age 85-89	−0.55	<.01
Age 90-94	−0.86	<.01
Age 95+	−1.37	0.08
Female	−0.34	<.01
Black (ref. group = white nonhispanic)	−0.40	<.01
Hispanic	−0.45	<.01
Asian	−0.34	0.08
Other Race	−0.35	<.01
Large Metro (ref. group = nonmetro)	0.22	0.02
Small Metro	0.07	0.02
% Black, 65+	−0.0014	0.44
% Below Poverty, 65+	−0.0140	0.02
Average Income, 65+	0.0121	<.01

## Mix and average medicare payments for specific services for surgical cataract episodes

In order to explore the possible extent of endogeneity bias in the estimation of the cataract episode model, we compared the average mix of specific services (by HCPCS code) provided to surgical and non-surgical episodes in the 1^st^ and 4^th^ quartiles of counties grouped by the ratio of the Medicare payment per episode for surgical episodes relative to non-surgical episodes. The specific services listed account for at least 1% of total claims per episode. The table lists services by the average Medicare payment per service from the most to least costly, and shows the share of claims attributed to each service and the ratio of the average Medicare payment for each service in the two groups of counties (Table 6).

**Table 6:** Mix of specific services for cataract episodes in low and high payment ratio counties

**HCPCS Code**	**Procedure Name**	**Counties in the 1**^**st**^**(lowest) Quartile of the Ratio of Medicare Payments for Surgical to Non-surgical Episodes**		**Counties in the 4**^**th**^**(highest) Quartile of the Ratio of Medicare Payments for Surgical to Non-surgical Episodes**		**Ratio of Medicare Payments in the 1**^**st**^**to the 4**^**th**^**Quartiles**
		**Pct. of Claims**	**Average Medicare Payment**	**Pct. of Claims**	**Average Medicare Payment**	
66982	Cataract surgery, complex	1.3%	$774	< 1%	--	--
66984	Cataract surgery w/iol, 1 stage	22.9	682	25.7	$569	1.20
66821	After cataract laser surgery	6.4	256	6.1	218	1.18
99244	Office consultation	1.5	137	1.5	122	1.12
92004	Eye exam, new patient	2.0	97	2.0	87	1.12
00142	Anesthesia for procedures on eye; lens surgery	12.7	81	13.7	62	1.32
92014	Eye exam & treatment	7.3	76	5.3	65	1.17
92135	Ophthalmic dx imaging	1.1	43	< 1%	--	--
99214	Office/outpatient visit, established patient	1.6	67	1.6	58	1.15
92012	Eye exam established pat	4.5	54	2.7	46	1.20
76519	Echo exam of eye	6.4	53	7.2	37	1.43
92136	Ophthalmic biometry	5.5	52	6.6	38	1.34
99213	Office/outpatient visit, established patient	2.3	43	2.2	38	1.13
Q1003	Ntiol category 3	1.6	40	1.6	40	1.00
